# Ten simple rules on writing clean and reliable open-source scientific software

**DOI:** 10.1371/journal.pcbi.1009481

**Published:** 2021-11-11

**Authors:** Haley Hunter-Zinck, Alexandre Fioravante de Siqueira, Váleri N. Vásquez, Richard Barnes, Ciera C. Martinez

**Affiliations:** 1 Berkeley Institute for Data Science, University of California, Berkeley, Berkeley, California, United States of America; 2 Bakar Computational Health Sciences Institute, University of California, San Francisco, San Francisco, California, United States of America; 3 VA Boston Healthcare System, Boston, Massachusetts, United States of America; 4 VA St. Louis Health Care System, St. Louis, Missouri, United States of America; 5 Energy and Resources Group, Rausser College of Natural Resources, University of California, Berkeley, Berkeley, California, United States of America; Dassault Systemes BIOVIA, UNITED STATES

## Abstract

Functional, usable, and maintainable open-source software is increasingly essential to scientific research, but there is a large variation in formal training for software development and maintainability. Here, we propose 10 “rules” centered on 2 best practice components: clean code and testing. These 2 areas are relatively straightforward and provide substantial utility relative to the learning investment. Adopting clean code practices helps to standardize and organize software code in order to enhance readability and reduce cognitive load for both the initial developer and subsequent contributors; this allows developers to concentrate on core functionality and reduce errors. Clean coding styles make software code more amenable to testing, including unit tests that work best with modular and consistent software code. Unit tests interrogate specific and isolated coding behavior to reduce coding errors and ensure intended functionality, especially as code increases in complexity; unit tests also implicitly provide example usages of code. Other forms of testing are geared to discover erroneous behavior arising from unexpected inputs or emerging from the interaction of complex codebases. Although conforming to coding styles and designing tests can add time to the software development project in the short term, these foundational tools can help to improve the correctness, quality, usability, and maintainability of open-source scientific software code. They also advance the principal point of scientific research: producing accurate results in a reproducible way. In addition to suggesting several tips for getting started with clean code and testing practices, we recommend numerous tools for the popular open-source scientific software languages Python, R, and Julia.

## Introduction

Creating functional, usable, and maintainable software is increasingly essential to open-source scientific research, especially in fields like bioinformatics [1,2]. Previous 10 simple rules papers have focused on open software development [3], robustness [4], usability [5], documentation [6], version control [7], and scientific programming [8]. Here, we add to this collection with tips on clean coding (standardized and clear writing styles) and test design (writing code to confirm program behavior) to further encourage high-quality scientific software development for academics building open-source scientific software packages. These suggestions are drawn from the authors’ experiences in developing software across academia, industry, and government laboratories and are predicated on the understanding that unit tests are fundamental to robust code development: They facilitate accurate modifications as well as new contributions and serve as a form of documenting intended behavior.

Clean coding, test design, and their execution are inconsistently introduced to academic researchers outside the software development domain. This results in a large variation in formal training for software development, sharing, and preservation [9]. Furthermore, the limited uptake of these tools could cause serious errors within software code, leading to inaccurate research results and paper retractions [10–13]. More broadly, estimated error rates in scientific publications, although not demonstrated to be higher than commercially developed software, have been estimated to range from approximately 3% for simple tasks to 14% for more complicated tasks [14]. Alarmingly, software popularity has not shielded against software bugs (flaws in program design that result in unwanted behavior): This is exemplified by the BLOcks SUbstitution Matrix (BLOSUM) matrix miscalculations, which persisted unnoticed for 15 years (although the miscalculations, ironically, improved search performance) [15].

In an effort to help scientific software developers prevent such issues, we propose 10 “rules” centered on 2 best practice components: clean code and testing. These 2 areas are relatively straightforward and can provide substantial utility relative to the learning investment. Existing institutional knowledge and growing community practice standards serve as evidence of their efficacy. Adopting clean code practices helps to standardize and organize software code in order to enhance readability and reduce cognitive load [16,17] for both the initial developer and downstream contributors [18]. Increasing readability while reducing cognitive load allows developers to concentrate on core functionality and reduce errors, while also exemplifying clean and inviting code for community open-source contributors.

Clean coding styles can also make software code more amenable to testing, for example, via unit tests that work best with modular and consistent software code. Modularity is a programming design technique that encourages the creation of functions to serve a single purpose, enabling interchangeability. Unit tests interrogate specific and isolated coding behavior to reduce coding errors and ensure intended functionality, especially as code increases in complexity [19]. Furthermore, unit test design provides benefits beyond checking for errors, including encouraging modularity and documenting code with example usage.

Although conforming to coding styles and designing tests can add time to the software development project in the short term, these foundational tools can contribute to improving the quality, usability, and maintainability of scientific software code for long-term research goals. This, in turn, can reduce the amount of time spent on a project later in its life cycle. Here, we suggest several tips for getting started and recommend tools for the popular open-source scientific software languages Python, R, and Julia (links included in Table 1 below) as scientific software developers tend to work in open-source over commercial software [9]. Creating a clean and standardized codebase is especially important in open-source software as there are often more diverse contributions (e.g., many different styles or levels of coding abilities) compared to commercial software. That said, we note that all of these rules can be applied to software development generally.

**Table 1:** Tools and resources in Python, R, and Julia for relevant clean and tested code rules.

Rule	Language	Purpose	Recommendation(s)	Alternative(s)
1	Python	Style guide	Google Python Style Guide and numpydoc	
		Style checker	pycodestyle and pylint	
		Style formatter	black	autopep8
	R	Style guide	tidyverse style guide	Google R Style Guide
		Style checker	lintr	formatR and styler
	Julia	Style guide	Julia style guide	
		Style checker	Julia linter in VSCode extension	Lint.jl and JuliaFormatter
3	Any	Parameter reduction	Parameter objects	
4	Any	Refactoring	Catalog of refactoring	
	Python	Refactoring	VSCode Python Refactoring extension	
5	Python	IDE*	VSCode	spyder and Gnome Builder
	R	IDE*	RStudio	VSCode and RKWard
	Julia	IDE*	VSCode	Juno
6	Python	Property-based testing	hypothesis	
	R	Property-based testing	hedgehog	
	Julia	Property-based testing	Checkers.jl and Test module	
8	Python	Test coverage	coverage	
	R	Test coverage	testCoverage	
	Julia	Test coverage	Coverage.jl	
9	Python	Test suite	snapshottest	
	R	Test suite	testthat	
	Julia	Test suite	testset	
10	Any	Automation	git hooks	
	Any	Automation	GitHub Actions	GitLab CI/CD

* IDE, integrated development environment.

### Rule 1: Choose a style guide—And stick with it

Each programming language has its own coding conventions, resulting in several acceptable ways to write code. When programs are written using a mixture of styles or employ no consistent style at all, the code can be difficult to read and absorb as a user or developer, even as an original developer who is returning to old code. To ensure standardization and maximize readability [16], adopt a style guide: a set of previously specified conventions on how to write code.

There are many style guides to choose from, but above all, consistency is key. When you choose one of many possible coding styles, apply that style throughout your code to ensure standardization. Google, for example, offers detailed style guides for several languages, including for Python and R. For Python documentation specifically, consider the numpy docstring guide. The tidyverse style guide is a good option for R users. Julia users can refer to the Julia style guide explicitly developed by that community.

In addition to passive style guide references, tools known as linters are available to check your adherence to a coding style automatically, highlighting any deviations from a chosen style. For Python, automated style checkers include pylint and pycodestyle, while tools such as black and autopep8 check and also reformat code to meet prespecified guidelines. For R, styler, formatR, or lintr can be used to automatically check coding style. For Julia, automated versions of style formatters exist in packages such as Lint.jl or are contained within extensions to integrated development environments (IDEs; see Rule 2) such as VSCode.

### Rule 2: Consider using an integrated development environment

Many of the tools referenced in Rule 1, as well as the testing frameworks discussed below (see Rule 6), are incorporated into IDEs such as VSCode or RStudio, to name a few. An IDE is a tool that generally makes it easier to develop software and usually consists of a source code editor, build automation tools, and a debugger with the potential to install additional extensions that further enhance functionality. Using an IDE makes it easy to write code, check style adherence, and run tests simultaneously.

In addition to providing a text editor, using an IDE will allow you to integrate many of the previously mentioned tools into one platform, making it even easier to follow style guidelines and clean coding practices. Some IDEs offer additional benefits including syntax highlighting, function suggestions, code outlines, automated refactoring, and easy file system access and editing via remote connections. Examples of IDEs that support multiple languages are VSCode and its open-source version VSCodium or language-specific IDEs like Spyder for Python, RStudio for R, and Juno for Julia.

### Rule 3: Reduce complexity when possible

In general, the more complex and less modular a piece of code is, the harder it will be to debug, understand, and adequately test. Modular design is based around the concept that each function or piece of code should have one purpose allowing the reuse in many areas of a script. For example, if a function does 2 things, try to split it into 2 functions. More concise and modular programming can make software easier to understand and debug by collaborators; code is, after all, read far more than it is written. Some formatting guidelines, like limiting line length, can help to make your code more readable [16] but need not be reduced to dogmatism. Other recommendations, like limiting the number of function arguments to a reasonable number (e.g., 5), can make your code easier for you or new members of your development team to absorb, modify, and troubleshoot. Parameter objects, a single argument object for commonly co-occurring groups of parameters, can help to limit the number of arguments per function while maintaining necessary inputs and functionality. Parameter objects can also improve the logical organization of multiple parameter inputs. Furthermore, limiting function length (e.g., 40 lines or less) can also help to reduce complexity and facilitate testing, usage, and expansion. Some of these limits on code length and complexity are automatically checked by linters mentioned in Rule 1, such as pylint in Python or lintr in R. JuliaFormatter integrates with previously referenced editors such as VSCode to enable this functionality in Julia.

### Rule 4: Refactor as needed

Refactoring is the process of restructuring your code without changing its interface—that is, rewriting the internals of functions without changing their inputs or outputs—often to improve its adherence to a set of best practices. Performing code refactoring frequently ensures that your software will be easier to understand, maintain, and expand while reducing the risk of introducing new errors.

Refactoring is often necessary for simple housekeeping over the course of development, for example, removing commented-out code or unused functions (e.g., dead code). Removing dead code reduces clutter and confusion in your program, making it easier to absorb. Refactoring may also be necessary to provide more substantial changes to the internal structure of a program to ensure that new features can be easily added. Modifying the internal structure can include changes like extracting a function to modularize a behavior and to avoid repeating code in several places. Another common refactoring involves grouping related functions into a single class. Automated analyses, such as tools (e.g., lintr) that highlight areas of high cyclomatic complexity, a metric measuring the number of separate pathways through your program’s logic, can help to highlight specific areas of your program that could benefit from refactoring.

When refactoring, do so incrementally, and always construct and run tests before implementing changes. Rerunning these tests after refactoring will ensure that functionality has been maintained. Moving in small increments will allow the changes to be rolled back easily if errors are introduced. While the amount and type of refactoring will depend on your program and application, an inspirational list of refactorings are available as a catalog. Following the measure of quality that “the true test of good code is how easy it is to change it,” quality refactoring should, above all, make your code easier to modify [20]. In addition to the catalog, several IDE extensions automate a subset of simple refactorings.

### Rule 5: Program defensively

All code may contain bugs, even for intended use cases, and additional issues may arise when the software is used in a way that the developers did not anticipate. Even the cleanest, most straightforward code can be used incorrectly. And as the codebase evolves, the definition of “correct” with respect to inputs, outputs, and use cases may well change. Therefore, to the extent possible, the developer should anticipate potential issues arising from different use cases and user error. This is called defensive programming or defensive design: proactively accounting for unexpected user interactions with the code [21]. Defensive programming principles allow your program to respond predictably in the face of unforeseen inputs.

Preconditions ensure that inputs to a function or code block have the expected data type, value ranges, or other qualities. These programmatic checks protect against unexpected user input. Similarly, postconditions ensure that the output of a given code block or function matches the value range and data type required for downstream analysis. There are multiple advantages to implementing these conditions and other defensive practices, such as exceptions, which enable users to self-correct by providing explanatory warning or error messages when an exception is encountered. Such checks furnish internal testing abilities and explicitly define expectations for input and output from commonly used functions.

### Rule 6: Follow established patterns for writing unit tests

Any good experiment should have checkpoints, validations, and controls. In order to determine if the code currently works as expected (and will continue to do so with future modifications), programmers use tests that are situated adjacent to the main codebase to automate the verification and validation of a program’s behavior. Unit tests check to verify the accuracy of specific pieces of code and are central to the growth of a production codebase and as such were expected to meet high-quality standards. Today, it is generally agreed that the primary purpose of unit tests is to protect against regressions or bugs that occur due to software modifications. Unit tests should be designed to satisfy 3 major principles. First, each unit test should evaluate only one behavior, focusing on the result of a code segment rather than details of implementation. Second, that behavior should be testable in isolation from other dependencies. Third, unit tests should be implemented such that their checks can be run with frequency for the sake of quick iteration, but without incurring substantial computational overhead [19].

There are additional broad guidelines for designing useful unit tests, but, to some degree, appropriate unit tests demand both creativity and subject matter knowledge on the part of the programmer. Basic prescriptions that apply to any codebase include straightforward test naming conventions that reduce coders’ cognitive load [17] and the use of specialized environments, called fixtures, to standardize test inputs and isolate targeted behavior from other dependencies [19]. As with coding style, consistency in unit test design is paramount. Certain language-specific tools, like pytest, inherently address both of these recommendations by requiring each testing function to begin with a standard naming prefix (e.g., “test_functionname”) and including functionalities for reusable unit tests. For example, using fixtures to specify predefined sample data to test functions each time your code is run enables consistency and simplicity.

Property-based testing can be used in conjunction with unit tests [22]. A property-based test passes a randomized input to a function and determines whether the output has expected properties. For instance, no matter what input you pass to a standard cosine function, the output should be bound to the range from −1 to 1. Since the input is randomized, property-based testing can find many problematic corner cases that a handcrafted unit test would miss. Some tools for property-based testing include Python’s hypothesis, R’s hedgehog, and Julia’s Checkers.jl.

### Rule 7: Create tests throughout the development cycle

Don’t wait until software completion to write tests! Waiting to write tests until the day after you develop a piece of code—or worse yet, when your program is at or near completion—will only provide time for you to forget the intended purpose of the code. It will also allow for an accumulation of unanticipated behavior, which may be difficult to disentangle. Writing clean code according to a consistent coding style can make it easier to write tests for your code throughout development. Testing from the beginning of the development cycle will allow for a strong foundation on which to expand your codebase and provide checks for base cases, boundary conditions, changes in functionality, and expansions of behavior as the code develops. This mindset also allows for (more) seamless continuous integration, the practice of frequently merging copies of code contributed by multiple developers to a single project. Designing software with testing in mind will also help reinforce a modular coding style, in line with many best practices, in order to ensure that software behavior is amenable to testing. In the extreme, test-driven development proposes writing tests before writing the target program code [23]. But at a minimum, writing tests when you encounter bugs will help to validate a fix and prevent such issues from reoccurring unexpectedly in the future. Finally, designing tests for each module throughout the development cycle provides documentation for code behavior and usage for future reference as the package increases in complexity.

### Rule 8: Ensure adequate testing coverage

How do you know when you have enough tests? Test coverage is a metric used to assess how much of your codebase is being tested by constructed unit tests. Ensuring that a line of code is covered in the context of a test helps guarantee that the code not only executes but will also execute with anticipated behavior. Calculating test coverage is one quantitative way to assess the sufficiency of your testing framework. You can calculate testing coverage, or the scope of your code that has been tested, in several ways, including the raw test coverage percentage, meaning the total lines of code in a piece of software executed by your unit tests divided by the total number of lines of code in your codebase. In general, the code coverage percentage may be easier to calculate, but branch coverage, the fraction of logical branches in your codebase that are executed by your tests, will give you a better sense of the tested logic. Optimal coverage estimates depend on the application and context of the codebase, but as a general rule of thumb, try to aim for at least 60% coverage [16].

That being said, it is important to remember that writing tests for scientific code can be difficult for several reasons, especially because of the intrinsic nondeterministic nature of exploring research questions [24]. Also, remember that tests also require maintenance, so ensure that tests are of high quality and adequate utility to merit inclusion. Tools for automatically calculating test coverage are available in many programming languages, such as the coverage package in Python, testCoverage package in R, and Coverage.jl in Julia.

### Rule 9: Set up end-to-end and regression testing

Unit testing and property testing (see Rule 6) help ensure that functions or simple compositions of functions work as intended. However, a full software program forms a complex system whose behavior may be difficult to predict, control, or test. Deep neural networks are a quintessential example: The mathematical operations that compose such a network (e.g., matrix multiplication and dot products) are easily tested, but the network’s aggregate behavior is not.

End-to-end tests address this problem. In the case of the neural network example, a simple end-to-end test might ask if metrics of the network’s performance (e.g., RMSE and AUC) meet expectations during initial tests or stay within a tolerance interval of previous performance when updates are made to any of the software layers that support a program—for example, an upgrade of the operating system or updates to base libraries. In particular, end-to-end tests can be coupled with property testing: If a model or simulation is not expected to be sensitive to variation in an input, this can be formally tested by running the model a number of times while varying that input.

End-to-end testing can also be accomplished by recording the outputs of a program and ensuring that they are constant over time. For instance, in particularly difficult testing situations relevant to app development, a screenshot of the app can be taken for each of its views. An end-to-end test can then compare the app’s user interface after a change and confirm that the views match. Similar techniques can be applied to the numerical outputs of programs. Packages for this include Python’s snapshottest and R’s testthat. Julia incorporates end-to-end testing into its base capacities by enabling the creation of custom testsets.

Finally, end-to-end tests are often slower to run than static analysis or unit tests. They also may require more complex testing frameworks and provide less useful feedback when they fail (as opposed to a unit test, which can pinpoint exactly which function input pair is problematic). For this reason, end-to-end testing may be brought into play later in the development cycle once the software is clean and well commented with a comprehensive test suite. However, since scientific software is often complex, end-to-end tests can provide a valuable verification that results have not unexpectedly changed while modifying the program.

### Rule 10: Automate your software testing workflow

By following all the previous rules, you will have a robust software package. Your code will be readable, consistent, and well covered by high-quality tests. However, coding styles and tests must be checked often; writing clean code and developing tests are habits that should start at the beginning of the software development cycle and continue throughout the process.

To ensure that your software will not be difficult to read or prone to break when you add new functionality or fix a bug, you need to run your test suite whenever you change part of your code. As mentioned in previous rules, tools are available to check coding style adherence (e.g., pylint, black, and JuliaFormatter) and run a suite of tests efficiently (e.g., pytest for Python, testthat for R, and testset for Julia). Initially, you may run these tools on an ad hoc basis, but automating linting and testing can add convenience and guarantee that these checks are consistently run.

When using online repositories such as GitHub [7], you can integrate these automated tools into continuous integration scripts that execute the tools upon a trigger event, like a code push, instead of running automated tools manually. This can be done using Git Hooks, a series of scripts that will run when an event occurs in your repository. For example, you can set your test suite to run when you commit new code, avoiding the necessity of running tests manually every time you modify your software. When using services such as GitHub or GitLab, these automated pipelines can be further integrated within the software workflow by using tools like GitHub Actions or GitLab CI/CD, in which testing and style checking can be additionally automated to run at specified times (e.g., daily at midnight). For an example of how to use GitHub Actions, please check GitHub’s documentation. There are also many online resources specific to Python, R, and Julia (including a community repository of prebuilt actions).

## Conclusions

Although adhering to consistent coding styles and developing tests may seem to divert attention from the main research goal, in fact, these practices help to advance the principal point of scientific undertakings: producing accurate results in a reproducible way. These rules are especially important in open-source software development: Clean code encourages a diversity of skill levels to contribute as maintainers; it also promotes more straightforward community code review protocols and assessment of code quality. Of course, community code review is a cornerstone of modern software development, whether the code in question is open source or proprietary [25–27]. Overall, these 10 simple rules will help to increase the clarity and robustness of your developed software.

The research environment’s increasing reliance on software tools reveals what can go wrong with small, and very human, mistakes. That being said, the conversation on software development for scientific research has shifted from “best” practices [28] to “good enough” practices [29]. Open-source scientific software is a collaborative endeavor requiring unique demands on researchers, and, therefore, standards should be adopted according to their appropriateness for your research community [30]. An individual or team of researchers should not strive to follow all best practices of software development, but rather strive to improve over time. The practices described here can become a natural part of your technical tool kit and rapidly add value in terms of quality and reproducibility in the scientific open-source software you produce.
